# Comprehensive pan-cancer analysis identifies PLAG1 as a key regulator of tumor immune microenvironment and prognostic biomarker

**DOI:** 10.3389/fimmu.2025.1572108

**Published:** 2025-04-10

**Authors:** Ruicheng Wu, Dengxiong Li, Shuxia Zhang, Jie Wang, Qingxin Yu, Dechao Feng, Ping Han

**Affiliations:** ^1^ Department of Urology, Institute of Urology, West China Hospital, Sichuan University, Chengdu, China; ^2^ Division of Surgery and Interventional Science, University College London, London, United Kingdom; ^3^ Research Core Facilities, West China Hospital, Sichuan University, Chengdu, Sichuan, China; ^4^ Department of Pathology, Ningbo Clinical Pathology Diagnosis Center, Ningbo, Zhejiang, China

**Keywords:** pan-cancer analysis, pleomorphic adenoma gene 1, biomarker, tumor-infiltrating cells, tumor microenvironment

## Abstract

**Background:**

The literature on the role of pleomorphic adenoma gene 1 (PLAG1) in malignant tumors is limited. This study aimed to perform pan-cancer analysis of PLAG1.

**Methods:**

The expression of PLAG1 was analyzed by Human Protein Atlas (HPA). The differential expression and prognosis of PLAG1 were analyzed based on TCGA pan-cancer data. The relationship between PLAG1 expression and tumor heterogeneity, stemness and immune infiltration was investigated. The enrichment analysis and biological function of PLAG1 in bladder cancer were analyzed.

**Results:**

The expression of PLAG1 was increased in a variety of tumors and significantly correlated with the prognosis of patients. Their expression levels were associated with key immune checkpoint genes (CD274, HAVCR2), immune infiltration and immune stimulation factors (CD48, CD27). In bladder cancer, functional enrichment analysis indicated that PLAG1 was involved in epidermal related processes and immune pathways. PLAG1 gene expression reduction can significantly inhibit the proliferation of bladder cancer cells.

**Conclusions:**

PLAG1 has the potential to be a prognostic marker and a potential therapeutic target for patients with malignant tumors.

## Introduction

Malignant tumors are currently the main cause of the overall burden of human diseases in the context of population aging (1, 2). Although traditional treatment methods, such as chemotherapy and radiotherapy, can inhibit the growth and spread of tumors, they are often accompanied by strong toxic side effects due to the inability to accurately distinguish tumor cells from normal cells (3, 4). In recent years, targeted therapy, as a treatment strategy specifically targeting key molecular markers in tumor cells, has gradually become a hotspot (5, 6). Compared to traditional treatments, targeted therapy offers higher specificity and lower toxic side effects, making it a promising avenue for future cancer treatment (7, 8). Therefore, in-depth study of the epigenetic mechanism of tumors is essential to understand its occurrence and development. Transcription factors affect the biological behavior of tumors by finely regulating gene expression. When their expression is abnormal or dysfunctional, they may accelerate tumor growth, invasion and metastasis (9). The research on transcription factors helps to provide new potential targets for cancer therapy (10, 11).

Pleomorphic adenoma gene 1 (PLAG1) is a zinc-finger protein located on chromosome 8q12 that was first identified in pleomorphic adenomas of the salivary gland (12). Under normal circumstances, PLAG1 expression is tightly regulated, and its oncogenic activation is significantly upregulated by chromosomal translocation and gene fusion, such as t (3; 8) (p21; q12) translocation leads to promoter exchange between PLAG1 and CTNNB1, which activates PLAG1 to promote salivary gland tumorigenesis (13). Recent studies have found that PLAG1 fusion can also exist in central nervous system embryonic tumors, which is a marker of high recurrence rate (14). On the one hand, PLAG1 expression in salivary gland tumors can be used as a marker of benign tumors (15). On the other hand, PLAG1 is involved in tumor progression by regulating downstream pathways such as IGF2 and Wnt, which are closely related to tumorigenesis in various solid malignancies (16, 17). Given the role of PLAG1 in malignant tumors, the association of PLAG1 with different tumor types has been rarely studied. We performed a comprehensive pan-cancer analysis using data from the Cancer Genome Atlas (TCGA) database. Our results highlight the prognostic relevance of PLAG1 in various tumor types, indicating its potential as a biomarker.

## Materials and methods

### Pan-cancer expression and prognostic analysis of PLAG1

We utilized the Human Protein Atlas (HPA) database (https://www.proteinatlas.org) to analysis the mRNA expression levels, subcellular localization, and single-cell analysis of PLAG1 in normal human tissues (18). TCGA pan-cancer dataset was retrieved from the UCSC database (https://xenabrowser.net/), employing the same research methods as in our previous studies (19). The expression levels of PLAG1 across various tumors in the TCGA were obtained using the “Gene_DE” module on the TIMER website (http://timer.cistrome.org/) (20). Additionally, the “Expression DIY” module on the GEPIA2 website (http://gepia2.cancer-pku.cn) was used to analyze PLAG1 expression in the TCGA and GTEx data (21). On the Sangbox platform (http://www.sangerbox.com), patients were categorized into high-expression and low-expression groups based on the median expression value of PLAG1 (22). The Cox proportional hazards regression model was employed, with disease-specific survival (DSS) and progression-free interval (PFI) as indicators to evaluate the prognostic significance of PLAG1. To assess the correlation between PLAG1 expression and clinical stage, gender, age, and other clinical characteristics, we applied the unpaired Wilcoxon rank-sum test, sign test, and Kruskal test.

### Analysis of tumor heterogeneity, stemness and mutation landscape

We utilized the Sangerbox platform to analyze the correlations between pan-cancer level PLAG1 expression and tumor stemness and heterogeneity, as well as the mutation landscape. The stemness indicators encompass six categories: differentially methylated probe-based stemness score (DMPss), DNA methylation-based stemness score (DNAss), enhancer element/DNA methylation-based stemness score (ENHss), epigenetically regulated gene expression-based stemness score (EREG.EXPss), epigenetically regulated DNA methylation-based stemness score (EREG-METHss) and RNA expression-based stemness score (RNAss). Reflective indicators of tumor heterogeneity include tumor mutation burden (TMB), mutant allele tumor heterogeneity (MATH), tumor purity, loss of heterozygosity (LOH), microsatellite instability (MSI), and homologous recombination deficiency (HRD). The samples were categorized into high-expression and low-expression groups based on PLAG1 expression levels, and the mutation landscape in bladder urothelial carcinoma (BLCA), low-grade glioma of the brain (LGG), and gastric adenocarcinoma (STAD) was illustrated.

### Analysis of immunorelated genes, immunoinfiltration, drug sensitivity and RNA modification

We utilized the R package “TCGAplot” to investigate the relationship between PLAG1 expression levels and a range of immune-related genes, which include immune checkpoint genes, chemokines, chemokine receptors, immunostimulants, and immunosuppressants (23). We calculated the correlations between PLAG1 gene expression levels and StromalScore, ImmuneScore and ESTIMATEScore. The Timer tool was employed to assess the correlation between PLAG1 expression and immune cell infiltration (20). Lastly, GSCALite (https://guolab.wchscu.cn/GSCA/) was used to evaluate the drug sensitivity of PLAG1 across different cancer types (24). The relationship between 44 genes involved in RNA methylation modification and PLAG1 expression was analyzed. PLAG1 promoter methylation levels in different types of cancer were assessed Using the UALCAN online tool (https://ualcan.path.uab.edu/) (25).

### Analysis of PLAG1-related biological function enrichment patterns in BLCA

We utilized RNA sequencing data from TCGA for BLCA. Based on the expression levels of PLAG1, we categorized BLCA patients into high-expression and low-expression groups for differential gene analysis. The differentially expressed genes were then subjected to gene set enrichment analysis (GSEA), which included both Gene Ontology (GO) enrichment analysis and Kyoto Encyclopedia of Genes and Genomes (KEGG) pathway analysis. The co-expression genes of PLAG1 in BLCA were identified using the R package “TCGAplot,” followed by GO functional enrichment analysis on these co-expression genes.

### Biological function of PLAG1

To explore the biological function of PLAG1 in bladder cancer cell lines, we used cell lines 5637 and T24 from ATCC center. Based on our previous overview of cell culture methods and real-time quantitative polymerase chain reaction (RT-qPCR) technology, we continued to conduct cell proliferation assays (19). Cells were incubated overnight in a humidified incubator at 37°C and 5% CO2 and subsequently analyzed in full-well, phase-contrast acquisition mode using the Incucyte Live Cell Assay system. Images were acquired every 8-12 h, and phase-area confluence was calculated with the Incucyte system. The resulting data were then normalized to day 0 to determine the relative phase object area, fold change, and expressed as mean ± SEM. The primer sequence utilized GAPDH as the internal control, with the following sequences: GAPDH: 5’-CTGGGCTACACTGAGCACC-3’ (forward) and 5’-TCCAAGTGGTCGTTGAGGGCAATG-3’ (reverse). PLAG1: 5’-GTTAAAGCCCCGCGATTGG-3’ (forward) and 5’-GGAACTGCCCAACTCCACT-3’ (reverse). Additionally, the sequences of small interfering RNA (siRNA) of PLAG1 were as follows: PLAG1 si-1 sense: 5’- GCUACUCAUUCUCCUGAGAAAdTdT-3’; PLAG1 si-1 antisense: 5’- UUUCUCAGGAGAAUGAGUAGCdTdT-3’. PLAG1 si-2 sense: 5’CCCACGUUUCCAUCAAGCUUUdTdT-3’; PLAG1 si-2 antisense: 5’- AAAGCUUGAUGGAAACGUGGGdTdT-3’; PLAG1 si-3 sense: 5’- GGUGAUUUGUCAGAAGUAAdTdT-3’; PLAG1 si-3 antisense: 5’- UUACUUCUGACAAAUCACCdTdT-3’. Control sense: 5’-UUCUCCGA ACGUGUCACGUdTdT-3’; Control antisense: 5’-ACGUGA CACGUUCGGAGAAdTdT-3’.

### Statistical analysis

Depending on the data’s normality and variance homogeneity, statistical analyses for continuous variables across three or more groups were performed using either a one-way ANOVA or the Mann-Whitney U test. The Student’s t-test was applied for comparing quantitative data between two groups. Data are reported as mean ± standard deviation. The R language version 4.4.2 was used. A p-value less than 0.05 was deemed to indicate statistical significance. Not significant (ns), P>0.05; *, P< 0.05; **, P<0.01; ***, P<0.001.

## Results

### Differential expression and clinical value

**Figure 1:**
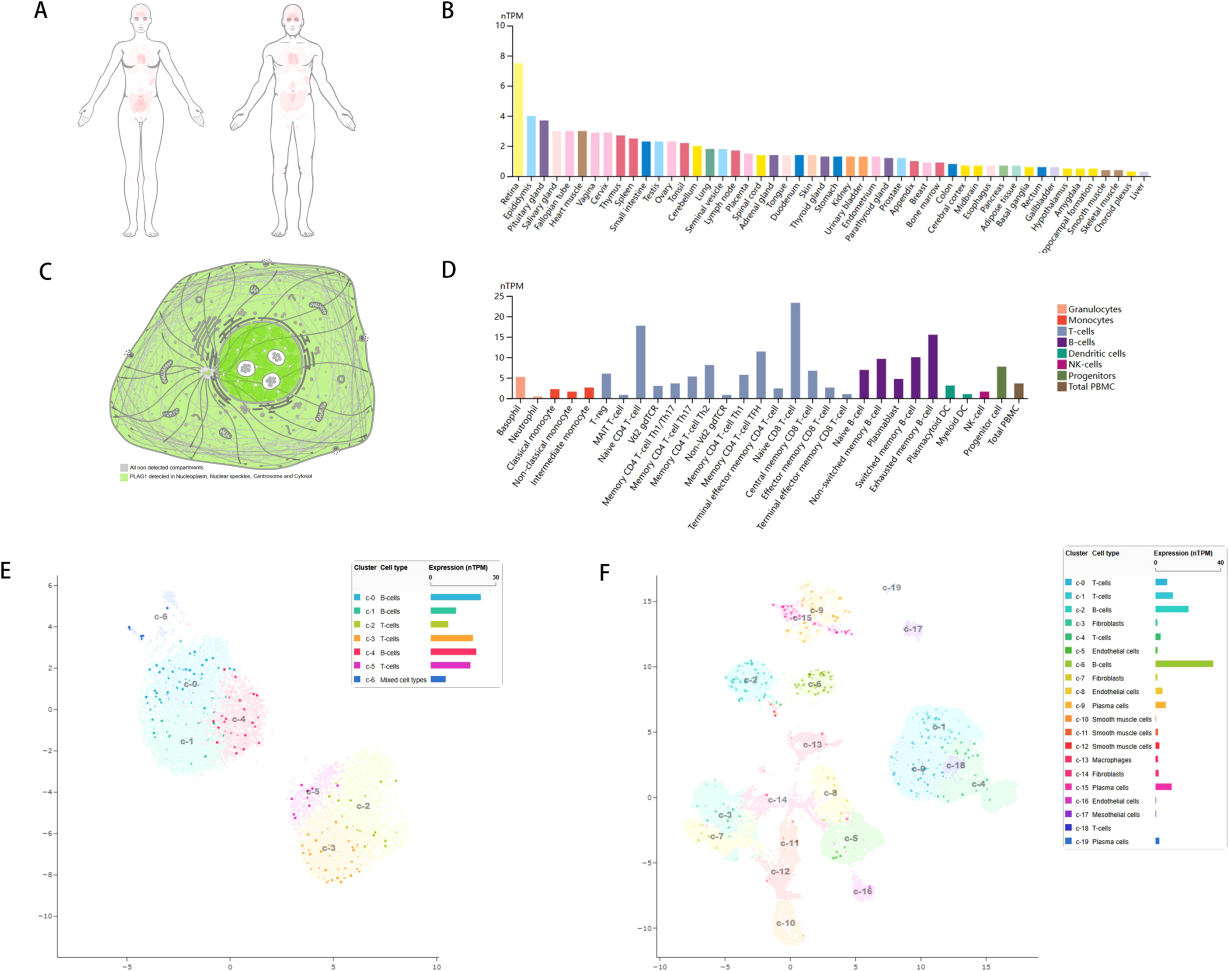
Gene expression of PLAG1. **(A)** Heat map of PLAG1 expression in human organs; **(B)** Expression of PLAG1 in various tissues in HPA database; **(C)** Subcellular localization of PLAG1; **(D)** PLAG1 expression in immune cells; **(E)** Single-cell analysis of TGS1 expression in various immune cells in lymph nodes; **(F)** Single-cell analysis of TGS1 expression in various immune cells in the thymus.

**Figure 2:**
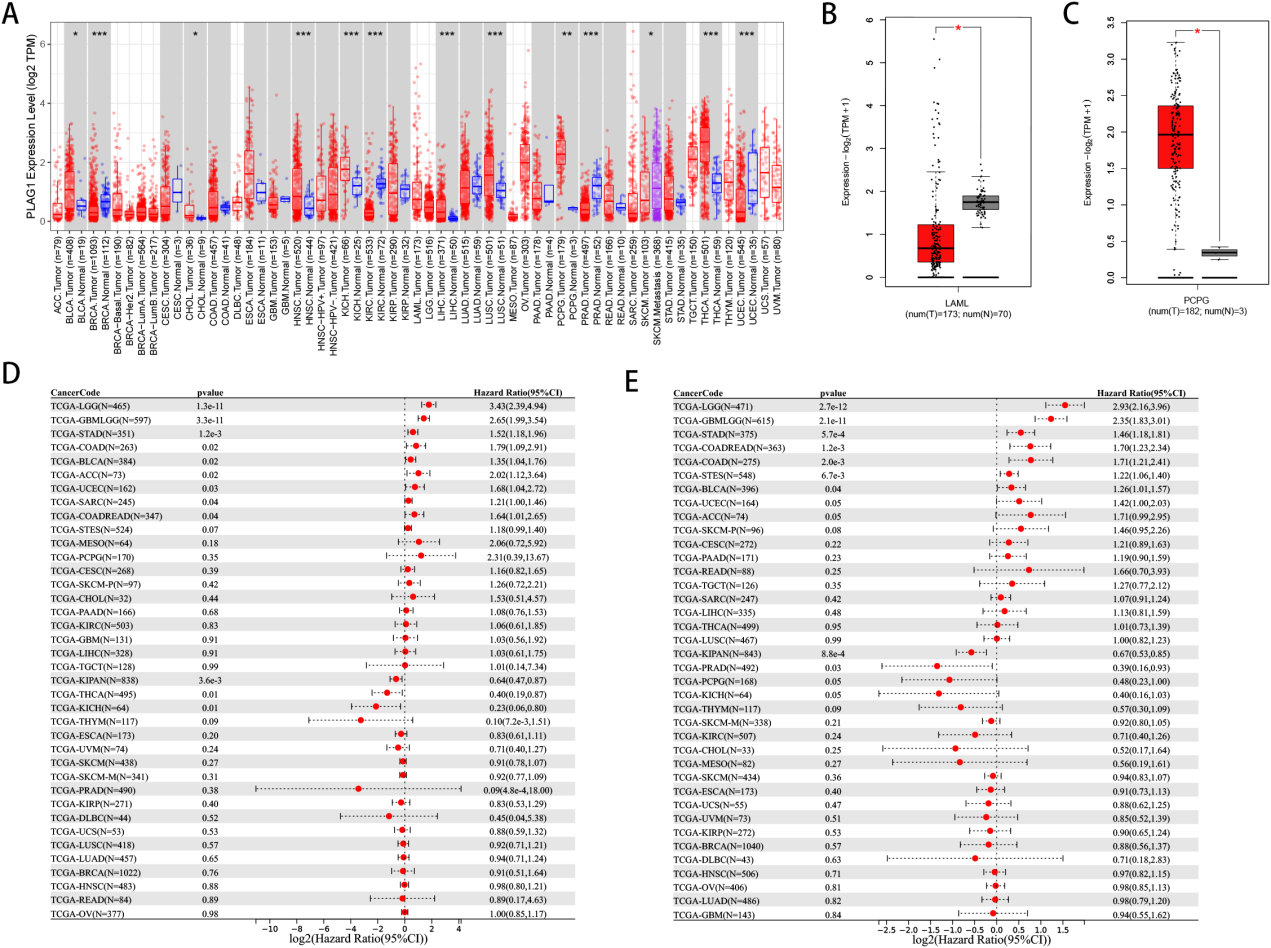
Pan-cancer expression and Prognosis of PLAG1. **(A)** Expression of PLAG1 gene in various tumors in TCGA. **(B)** expression of PLAG1 in LAML in GTEx and TCGA; **(C)** expression of PLAG1 gene in PCPG in GTEx and TCGA; **(D)** Pan-cancer analysis of PLAG1 for DSS; **(E)** Pan-cancer analysis of PLAG1 for PFI. *, P< 0.05; **,P<0.01; ***, P<0.001.

To investigate the expression levels of PLAG1 in various normal human tissues, we utilized the HPA database for our analysis. The expression of PLAG1 across human organs and the subcellular localization of the protein are illustrated in **Figures 1A–C**. Our findings indicate that PLAG1 is predominantly expressed at high levels in T cells and certain subsets of B cells (**Figure 1D**). Similar conclusions were reached through single-cell analysis targeting the thymus and lymph nodes (**Figures 1E, F**), suggesting that PLAG1 may play a role in regulating immune cell activation. Our results demonstrate that PLAG1 is highly expressed in eight types of tumor tissues (**Figure 2A**); however, its expression in tumor tissues is lower than that in normal tissues in uterine corpus endometrial carcinoma (UCEC), kidney renal clear cell carcinoma (KIRC), breast invasive carcinoma (BRCA) and prostate adenocarcinoma (PRAD) (**Figure 2A**). In the combined analysis of GTEX and TCGA data, PLAG1 highly expressed in tumors in pheochromocytoma and paraganglioma (PCPG), while its expression level in the bone marrow or peripheral blood of acute myeloid leukemia (LAMA) patients is lower than that in healthy controls (**Figures 2B, C**).

Our analysis reveals a significant correlation between PLAG1 and DFS (**Figure 2D**) as well as PFI (**Figure 2E**) across multiple cancer types. It is noteworthy that LGG, glioma (GBMLGG), STAD, BLCA, and colon adenocarcinoma/rectal adenocarcinoma (COAD/READ) exhibit significant correlations with these prognostic indicators (**Figures 2D, E**). Additionally, PLAG1 demonstrates varying correlations with the clinical characteristics of different tumors, including T stage, N stage, M stage, and clinical stage (**Supplementary Figures S1A–D**). Furthermore, among the 11 tumor types analyzed, the PLAG1 mRNA expression shows a significant correlation with age, with 4 tumor types exhibiting a positive correlation and 7 showing a negative correlation (**Supplementary Figure S1E**).

### Relationship of PLAG1 with tumor heterogeneity, stemness and gene mutation

**Figure 3:**
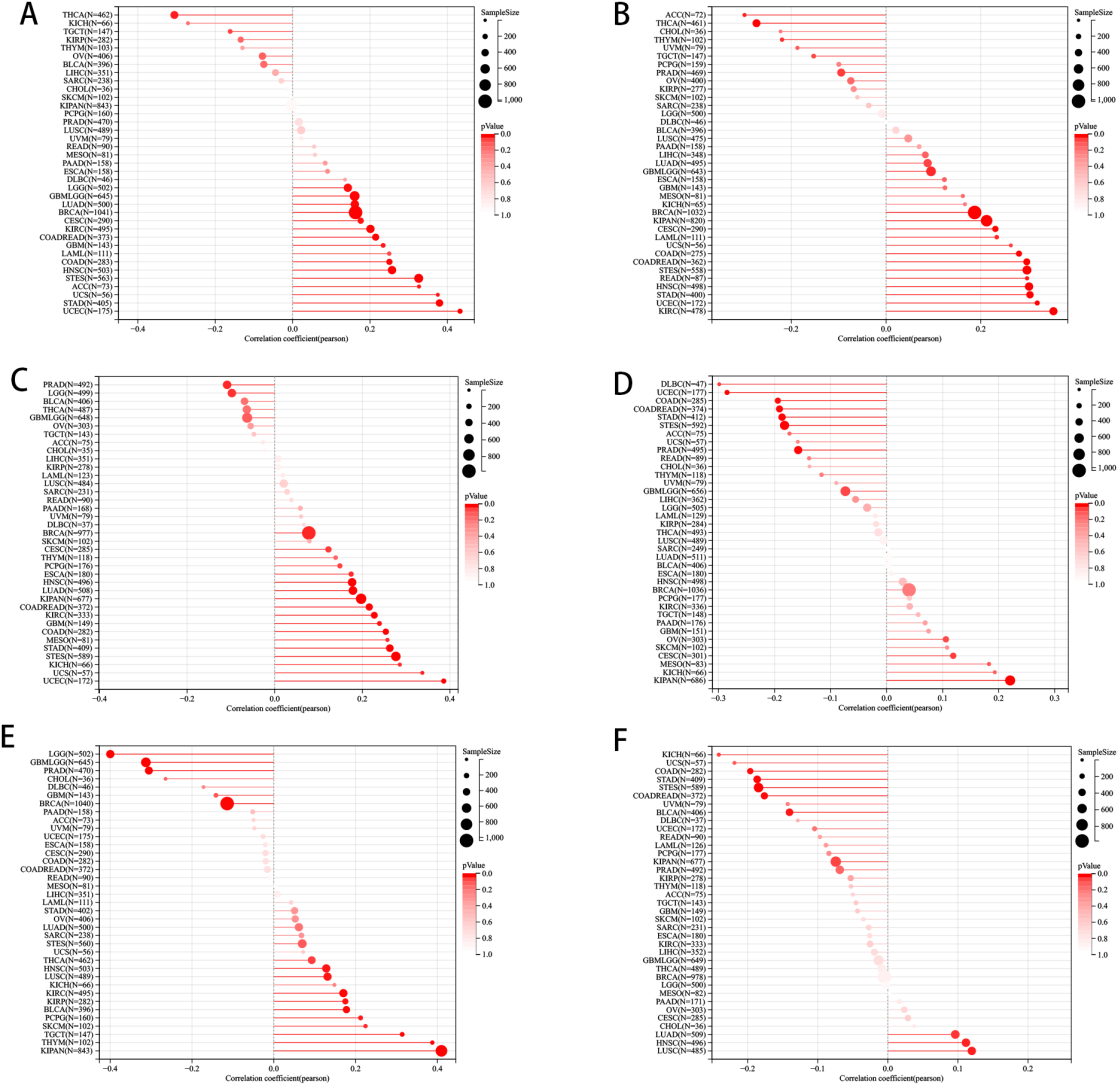
The pan-cancer Spearman analysis of tumor heterogeneity and PLAG1 expression. **(A)** The correlation between HRD and PLAG1 level; **(B)** The correlation between LOH and PLAG1 level; **(C)** The correlation between MATH and PLAG1 level; **(D)** The correlation between MSI and PLAG1 level; **(E)** The correlation between TMB and PLAG1 level; **(F)** The correlation between tumor purity and PLAG1 level;.

**Figure 4:**
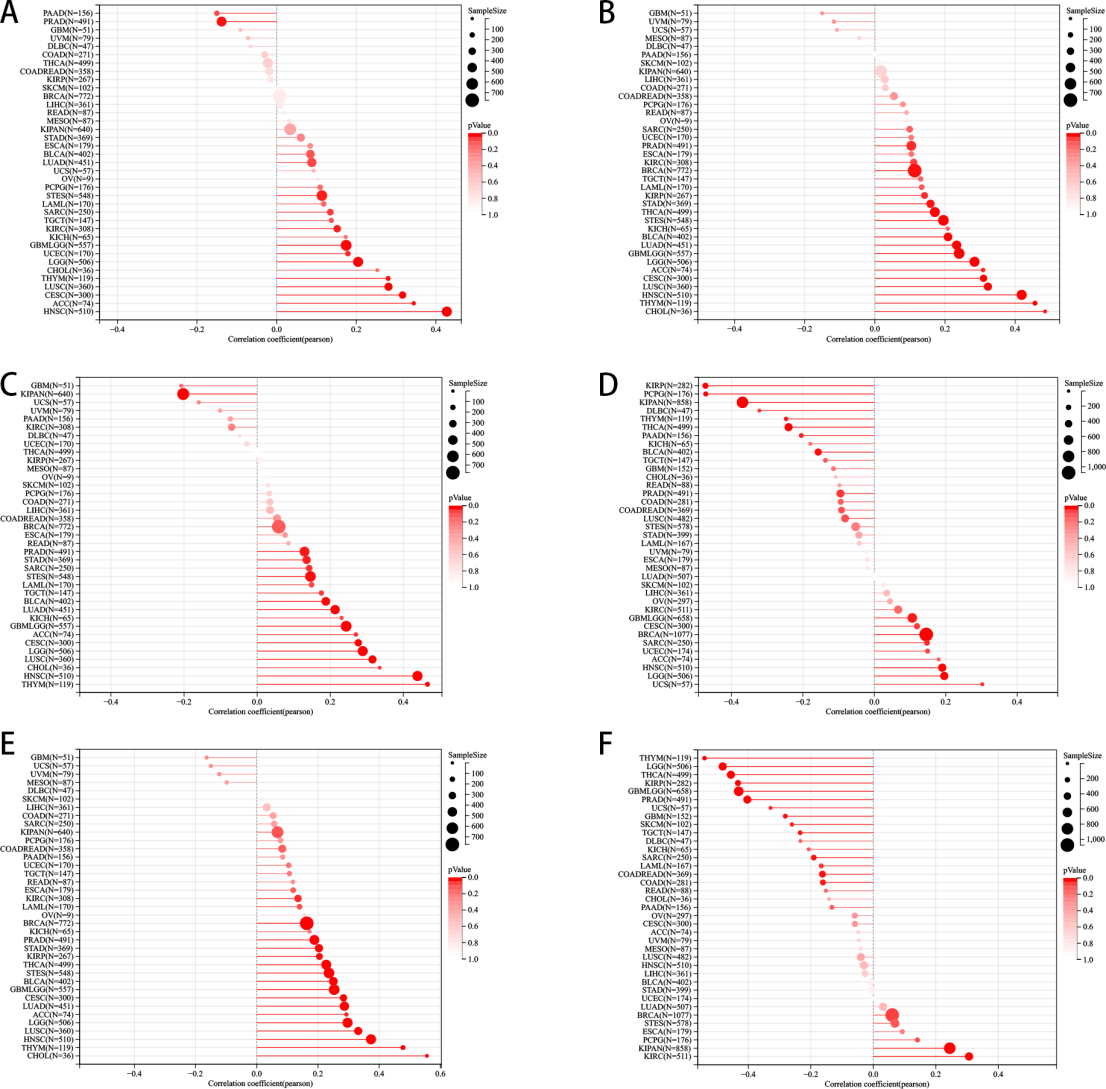
Pan-cancer Spearman correlation analysis between PLAG1 expression and stemness indices. **(A)** Correlation between PLAG1 expression and DMPss; **(B)** Correlation between PLAG1 expression and DNAss; **(C)** Correlation between PLAG1 expression and ENHss; **(D)** Correlation between PLAG1 expression and EREG. EXPss; **(E)** Correlation between PLAG1 expression and EREG-METHss; **(F)** Correlation between PLAG1 expression and RNAss.

We delved deeper into examining the relationship between PLAG1 expression levels and tumor heterogeneity and stemness. A notable correlation was discovered between PLAG1 expression levels and HRD status in 16 tumors (**Figure 3A**). Additionally, a positive association between PLAG1 expression and LOH was identified in 14 tumors (**Figure 3B**). Regarding MATH, it was observed that PLAG1 mRNA expression had a negative correlation with 2 tumor types (**Figure 3C**). The research results indicated a significant connection between PLAG1 expression and MSI in 11 tumors, encompassing COAD and STAD (**Figure 3D**). However, across 15 tumors, there was a significant correlation between tumor purity with PLAG1 expression (**Figure 3E**). Additionally, in eight tumors, PLAG1 expression was found to be correlated with TMB (**Figure 3F**). In the assessment of tumor stemness, a notable positive correlation was found between PLAG1 expression levels in LGG and GBMLGG and all six types of tumor stemness (**Figures 4A–F**).

**Figure 5:**
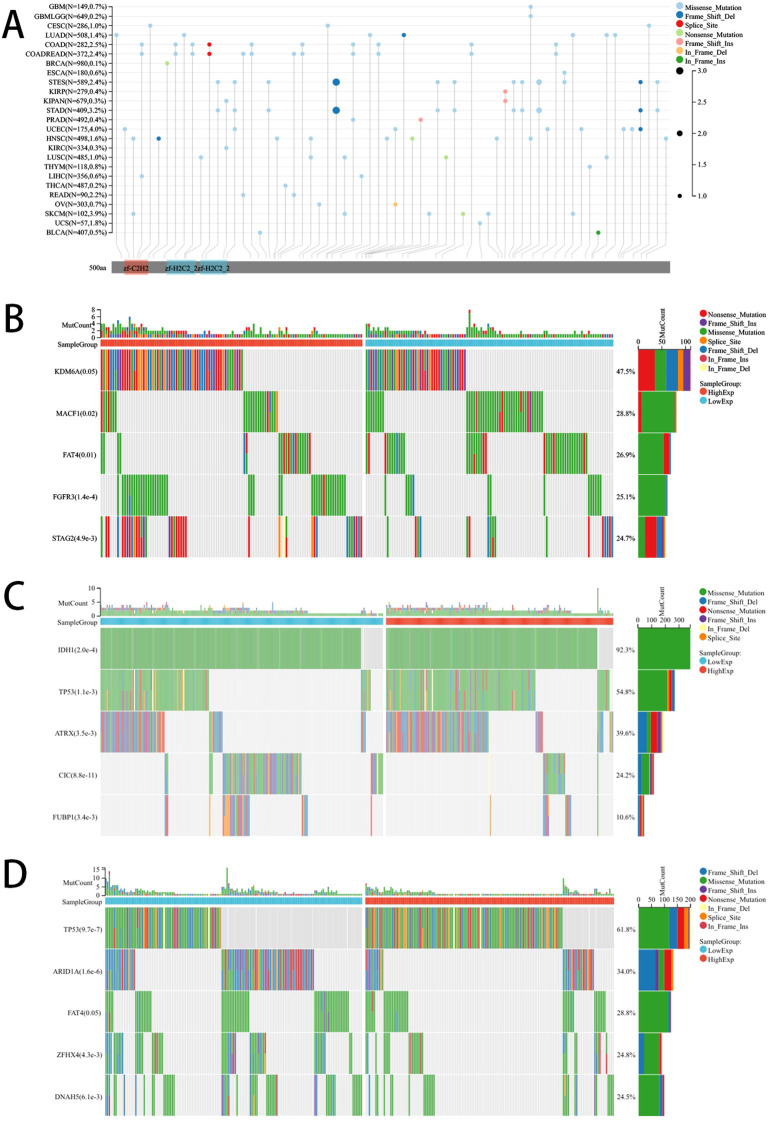
Mutation landscape of PLAG1. **(A)** Mutation landscapes of PLAG1 for pan-cancer; **(B)** The top 5 mutation genes between high and low-expression of PLAG1 in BLCA patients; **(C)** The top 5 mutation genes between high and low-expression of PLAG1 in LGG patients; **(D)** The Top 5 mutation genes between high and low-expression of PLAG1 in STAD patients;.

Tumor gene mutations are known to play a pivotal role in the growth and spread of tumors. This study specifically examined the mutation patterns of PLAG1 across various types of tumors. By comparing the mutation profiles between the high-expression and low-expression groups of PLAG1, we were able to identify genes that were significantly mutated. Our analysis revealed mutations in MACF, FAT4, and FGFR3 in BLCA, as well as mutations in IDH1 and TP53 in LGG. Additionally, TP53 mutations were also observed in STAD (**Figures 5A–D**).

### Relationship between PLAG1 expression with Immunorelated genes, drug sensitivity and RNA modification

**Figure 6:**
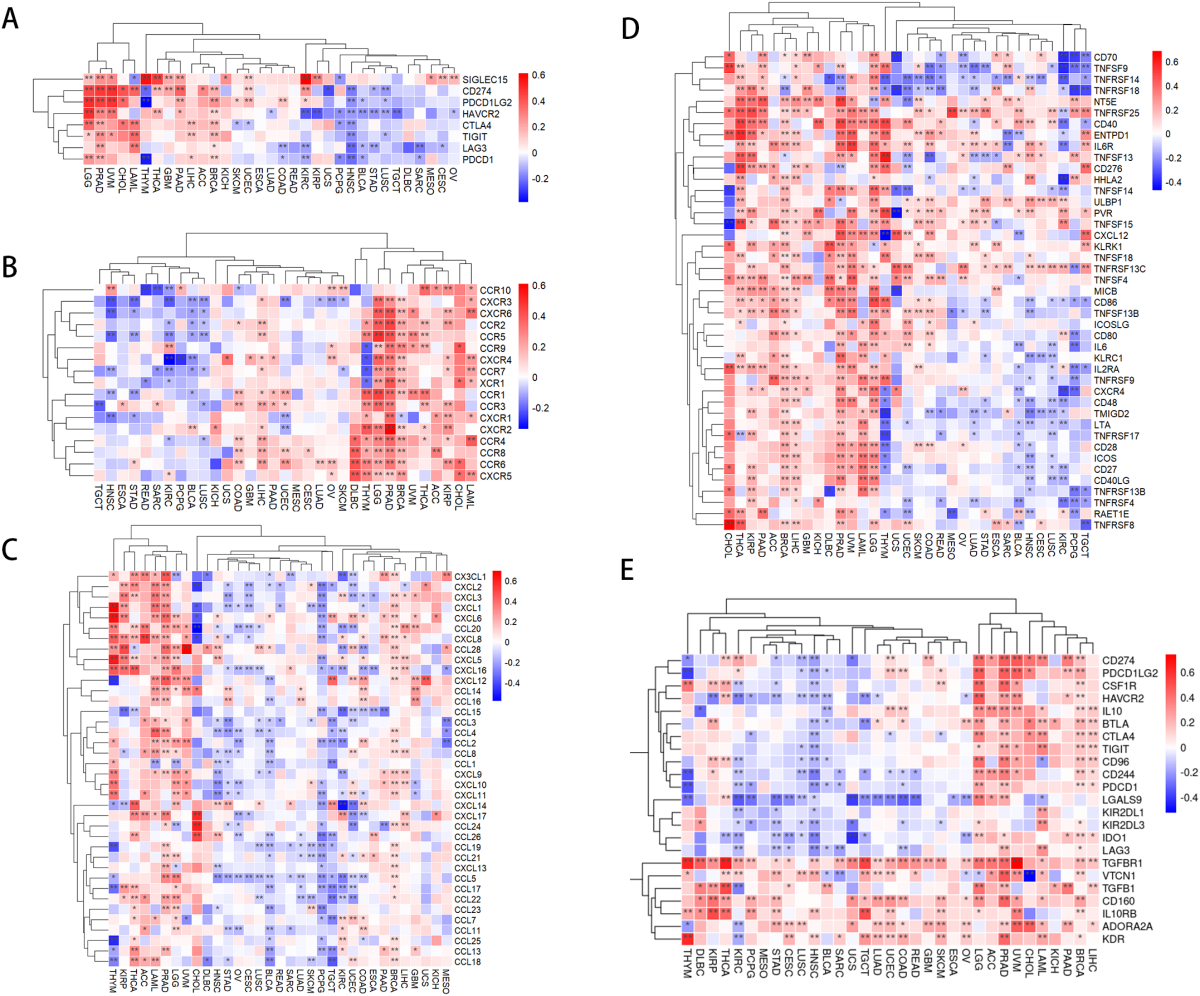
Correlation between PLAG1 expression and immune-related genes **(A)** correlation between PLAG1 expression and immune checkpoint genes; **(B)** correlation between PLAG1 expression and chemokines; **(C)** correlation between PLAG1 expression and chemokine receptors; **(D)** correlation between PLAG1 expression and immunostimulatory molecules; **(E)** Correlation between PLAG1 expression and immunosuppressive molecules. *, P< 0.05; **,P<0.01;.

Our study indicates that the expression levels of PLAG1 across various cancer types are associated with multiple immune-related genes. In the analysis of immune checkpoint (IC) genes (**Figure 6A**), PLAG1 exhibits a positive correlation with key IC genes such as HAVCR2 and CD274 in cancer types like LGG and PRAD, while showing a negative correlation in head and neck squamous cell carcinoma (HNSC) and STAD. Regarding chemokines and their receptors (**Figures 6B, C**), PLAG1 is significantly positively correlated with chemokines such as CXCL9, CXCL10, and CCL5, as well as their receptors (including CCR5, CXCR3, and CXCR4) in PRAD and BRCA. These chemokines are typically involved in the recruitment of T cells, NK cells, and macrophages. Further analysis of immune-stimulating factors (**Figure 6D**) revealed that in tumors such as LGG, PRAD, and BRCA, the expression of PLAG1 was significantly positively correlated with immune-stimulating factors like CD48, CD27, and TMIGD2, which may enhance anti-tumor immune responses. In contrast, in KIRC and lung squamous cell carcinoma, PLAG1 was negatively correlated (**Figure 6D**), suggesting that PLAG1 may exhibit immune regulatory heterogeneity across different cancer contexts. Additionally, concerning immunosuppressive gens (**Figure 6E**), PLAG1 was significantly positively correlated with TIGIT, CD96, and CD244 in LGG, uveal melanoma, and liver hepatocellular carcinoma, indicating that it may facilitate the formation of a tumor immune-suppressive microenvironment.

**Figure 7:**
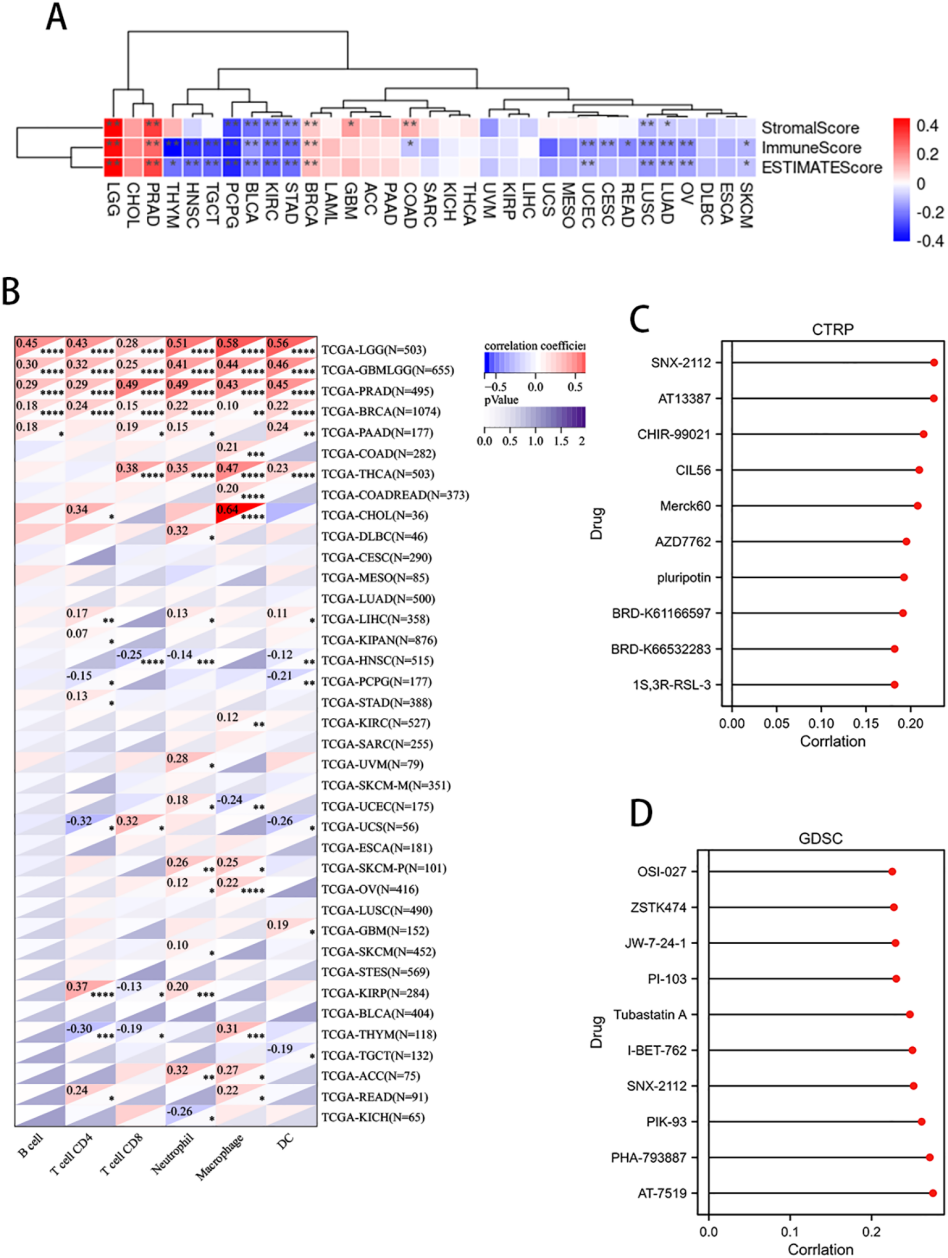
Correlation between PLAG1 expression and immune infiltration and drug sensitivity analysis **(A)** correlation between PLAG1 expression and immune infiltration score; **(B)** Correlation between PLAG1 expression and immune infiltrating cells detected by TIMER; **(C)** correlation between PLAG1 expression and drug sensitivity in CTRP database (top 10); **(D)** Correlation between PLAG1 expression and drug sensitivity in GDSC database (top 10). *, P< 0.05; **,P<0.01; ***, P<0.001;****, P<0.0001.

The expression of PLAG1 is significantly negatively correlated with StromalScore, ImmuneScore, and ESTIMATEScore in LGG, BLCA, KIRC, and STAD (**Figure 7A**). Further analysis of immune cell infiltration revealed that PLAG1 is positively correlated with the infiltration levels of B cells, T cells, and dendritic cells (DC) in cancer types such as LGG, GBMLGG, PRAD, and BRCA, while it is negatively correlated with the infiltration of certain immune cells (such as CD8+ T cells and dendritic cells) in cancer types including KIRC and LIHC (**Figure 7B**). This finding suggests that PLAG1 may exert different regulatory effects on immune cell infiltration across various tumor types.

**Figure 8:**
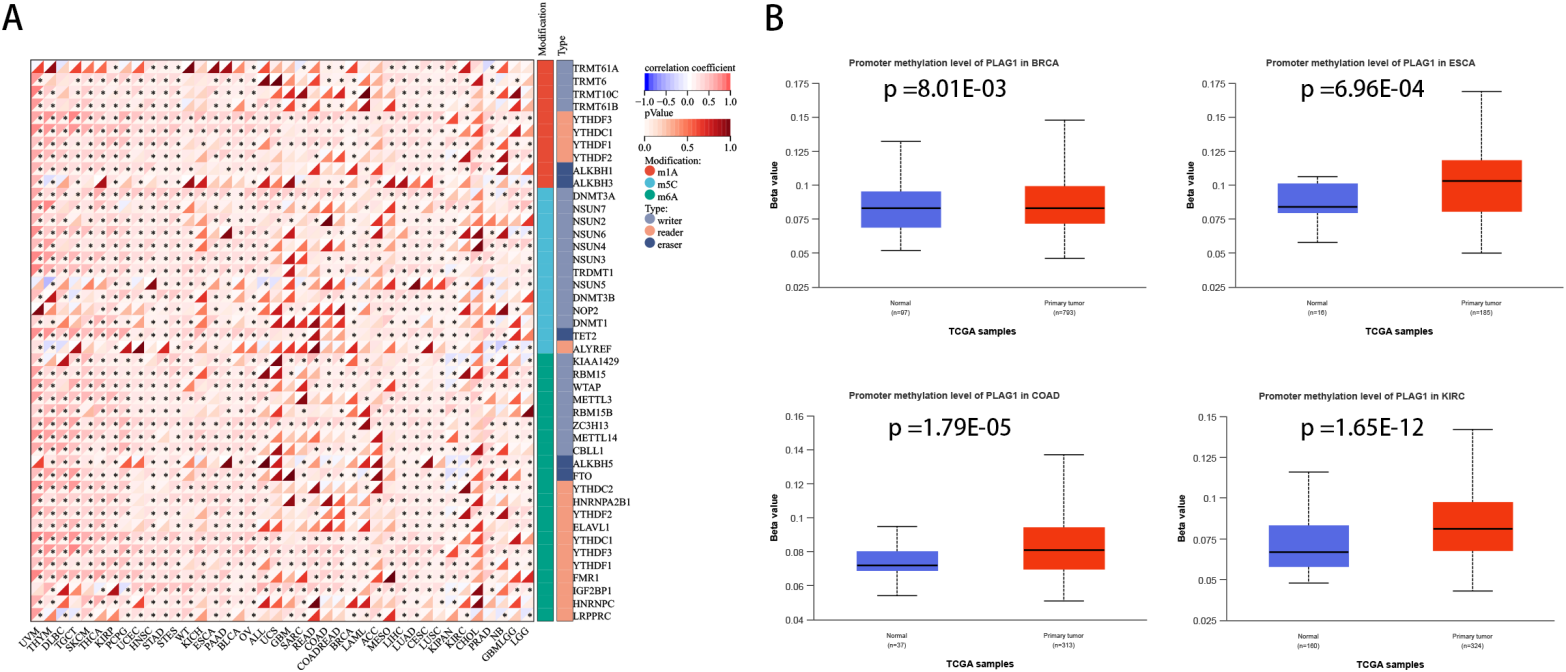
Correlation between PLAG1 expression and RNA modification and DNA methylation **(A)** Correlation between PLAG1 expression and RNA modification; **(B)** promoter methylation levels in different tumor types and corresponding normal tissues. *, P<0.05.

The correlation analysis between PLAG1 and drug sensitivity demonstrates that, within the cancer therapeutics response portal (CTRP) and genomics of drug sensitivity in cancer (GDSC) databases, PLAG1 expression is significantly correlated with the sensitivity to various anticancer drugs, including pluripotin, tubastatin A, PIK-93, and AT-7519 (**Figures 7C, D**). This suggests that PLAG1 may influence the response of cancer cells to specific targeted therapies or chemotherapy agents. Furthermore, our research findings indicate that in most tumors, PLAG1 is positively correlated with the expression of m1A, m5C, and m6A modifications (**Figure 8A**). We also assessed the promoter methylation levels of PLAG1 across different cancer types. Notably, in the BRCA, esophageal carcinoma (ESCA), COAD, and KIRC, the promoter methylation levels of PLAG1 were significantly elevated in tumor tissues (**Figure 8B**).

**Figure 9:**
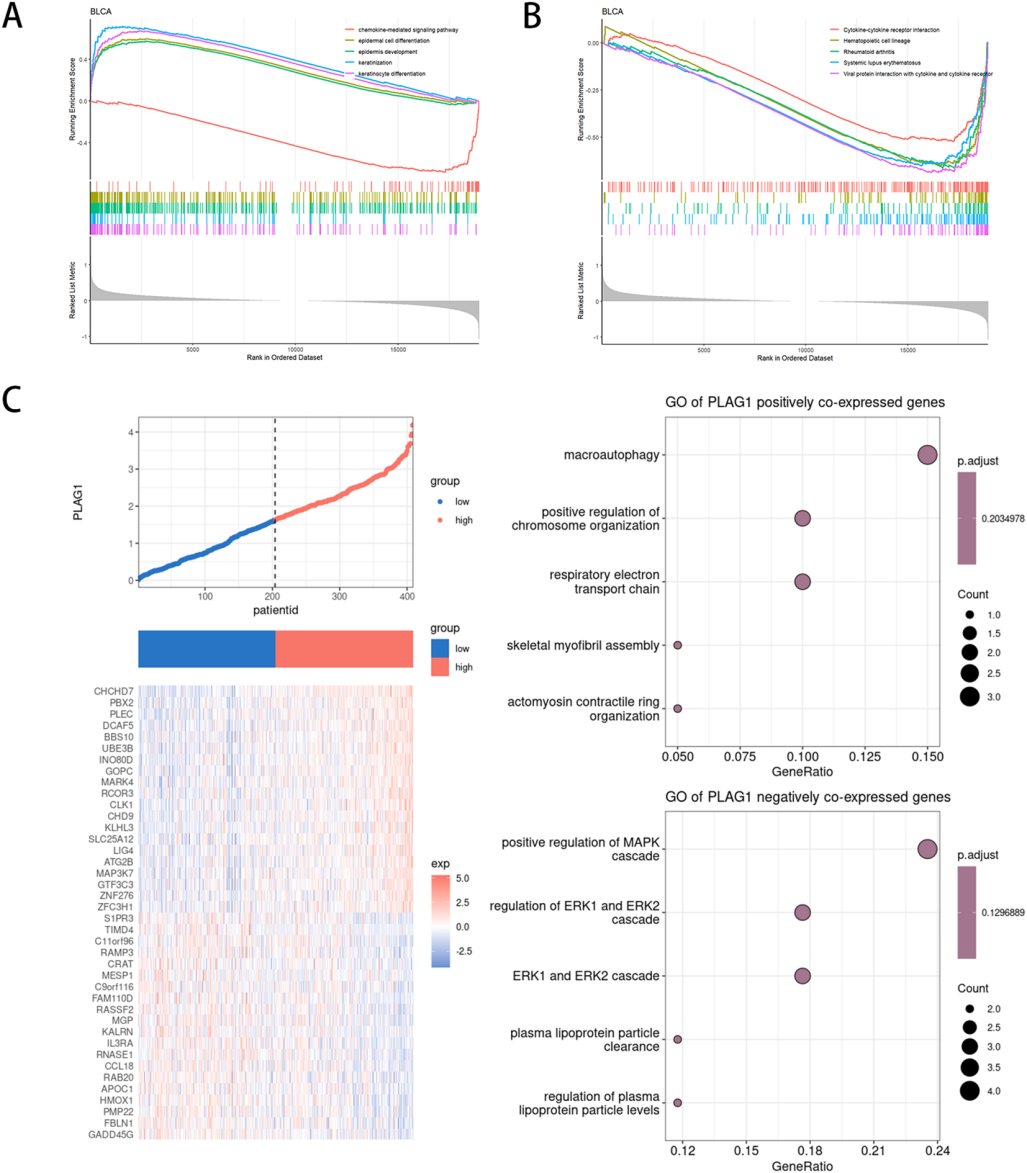
Functional enrichment analysis of PLAG1 in BLCA **(A)** GSEA based on GO enrichment analysis in BLCA patients;**(B)** GSEA based on KEGG enrichment analysis in BLCA patients;**(C)** Co-expression analysis of PLAG1 in BLCA.

We analyzed the differentially expressed genes between the high-expression and low-expression groups of PLAG1 in BLCA (**Supplementary Figure S1F**). The potential biological functions of PLAG1 in BLCA were explored using GSEA. In the GO analysis, the high-expression group of PLAG1 was enriched in epidermal-related processes and may have a potential connection to epithelial-mesenchymal transition in tumors (**Figure 9A**). Additionally, the chemokine-mediated signaling pathway was enriched in the low-expression group of PLAG1. In the KEGG analysis, it was found that the low-expression group of PLAG1 was enriched in immune-related signaling pathways (**Figure 9B**). Subsequently, we analyzed the co-expressed genes of PLAG1 in BLCA and conducted GO analysis on its positively and negatively co-expressed genes, respectively. The positively co-expressed genes of PLAG1 were mainly enriched in macroautophagy and the respiratory electron transport chain, while the negatively co-expressed genes were significantly enriched in pathways such as the positive regulation of the MAPK cascade and the regulation of ERK1 and ERK2 cascades (**Figure 9C**).

### Cell proliferation

**Figure 10:**
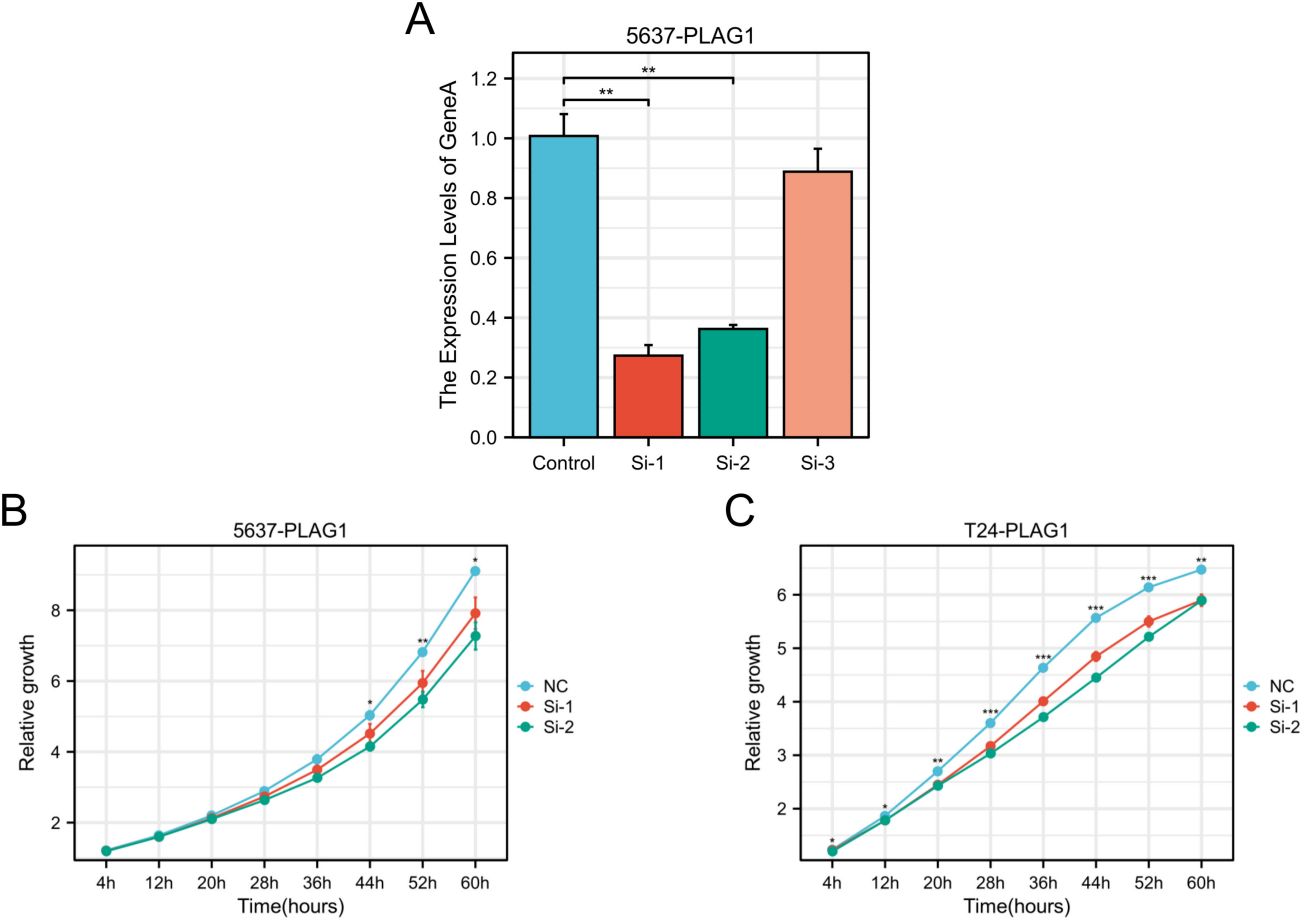
Effect of PLAG1 on the biological behaviors of BLCA. **(A)** RT–qPCR results of PLAG1 siRNAs on 5637; **(B)** Proliferation of PLAG1 siRNAs on 5637; **(C)** Proliferation of PLAG1 siRNAs on T24. *, P< 0.05; **,P<0.01; ***, P<0.001.

The use of RT-qPCR assay revealed that PLAG1 siRNA1 and siRNA2 effectively decreased the expression of PLAG1 mRNA in 5637 cells (**Figure 10A**). Following these findings, siRNA1 and siRNA2 were chosen for further experiments, demonstrating a significant inhibition in the proliferation of both 5637 and T24 cells (**Figures 10B, C**).

## Discussion

The application of multi-omics technology enables the identification of key driver genes in tumors and facilitates the discovery of therapeutic targets beyond conventional surgical treatment options (26, 27). With the help of multi-omics technology, the elucidation of the molecular characteristics of tumors can help us to identify the key driver genes of tumors and find targeted therapeutic targets (28, 29). PLAG1 is closely related to benign salivary gland tumors and stromal tumors, and it can be used as a diagnostic marker for pleomorphic adenoma (30). Gene fusions such as NDRG1-PLAG1 and TRPS1-PLAG1 have also been reported in rare diseases such as syringoma chondroid (31).In addition, PLAG1 rearrangement is seen in uterine myxoid leiomyosarcoma (approximately 25% of cases) (32). In developmental regulation, PLAG1 maintains the quiescent state of hematopoietic stem cells (HSC) by inhibiting protein synthesis, thereby enhancing the stemness of HSC (33). Meanwhile, PLAG1 cooperates with USF2 to regulate MSI2 expression, constituting the core of HSC transcriptional network (34). PLAG1 deletion is associated with Silver-Russell syndrome, which is characterized by fetal growth restriction and facial dysmorphology (35). Moreover, knockout of PLAG1 in mouse models leads to growth retardation, reduced fertility, and hearing impairment (36, 37). Twenty years ago, some studies pointed out that abnormal expression of PLAG1 may lead to uncontrolled cell proliferation, which was consistent with the mechanism of the occurrence of a variety of malignant tumors (38). Zatkova et al. found that PLAG1 gene amplification was related to the occurrence of hepatoblastoma, and later, some studies also linked PLAG1 with malignant tumors (39). Later, studies have also explored the relationship between PLAG1 and malignant tumors, and found that its expression is related to the invasiveness and prognosis of a variety of malignant tumors, including LIHC (40), KIRC (41) and invasive pituitary adenoma (42). Therefore, we performed a systematic pan-cancer analysis of PLAG1, aiming to explore its expression pattern, genetic alterations in different tumor types and its relationship with clinical prognosis.

Based on data from TCGA, we found that PLAG1 was significantly upregulated in eight types of tumor tissues compared to normal tissues. This includes malignant tumors with high invasiveness, such as BLCA and LIHC. Recent studies have also indicated that high expression levels of PLAG1 in LIHC are associated with poor prognosis (43). The prognostic value of PLAG1 was investigated by analyzing tumor DFS and PFI. Our study revealed an association between PLAG1 and poor prognosis in specific tumors, such as GBMLGG, STAD and BLCA. This aligns with Luo et al.’s study, which demonstrated that a circular RNA activated PLAG1 expression, enhancing survival of gastric cancer cells and contributing to malignant phenotype and chemoresistance (44). In the Pan-kidney cohort, there was a notable correlation between PLAG1 expression levels and both T stage and N stage. This implies that PLAG1 may be subject to epigenetic regulation during tumor development, highlighting its potential as a predictive marker for clinical staging. In addition, our study also highlights the correlation of age with PLAG1 expression, aging is considered an important factor in tumorigenesis, and the accumulation of DNA damage and increased levels of inflammation create a favorable environment for tumor cells to evade immune surveillance (45, 46).

Tumor heterogeneity refers to the existence of populations of cancer cells with different genetic characteristics within a tumor. This allows specific cancer cell populations to gain certain proliferative advantages in the tumor microenvironment (TME) (47, 48). We examined the correlation between PLAG1 expression and tumor heterogeneity by analyzing six indicators. The results showed that PLAG1 expression level was significantly correlated with HRD in fifteen tumors, one of which showed a positive correlation. High HRD tumors usually have strong genomic instability and often have high TMB, which increases the production of tumor neoantigens and enhances the sensitivity of immunotherapy (49, 50). Therefore, we hypothesized that alterations in PLAG1 expression levels may affect HRD and thus patient response to therapy. Jin et al. found that PLAG1 induces GDH1 expression in lung cancer, and GDH1 activates CamKK-AMPK signaling through α-KG, a product of glutamine metabolism, to improve tumor anti-apoptosis (51). However, excessive glutamine depletion may suppress T-cell activity (52). Previous studies have also pointed out that α-KG affects T cell differentiation and may regulate immune suppression or activation (53, 54). Although there is no clear experimental data to point out the role of PLAG1 in tumor immunotherapy, our results also found that PLAG1 expression was strongly correlated with multiple immune checkpoint genes and correlated with tumor immune infiltration, so we speculated that PLAG1 may affect the efficacy of immunotherapy by regulating downstream signaling pathways. There was a negative correlation between PLAG1 expression and MSI in seven tumors, and tumors with high MSI tended to be radioresistant (55). There may also be a potential relationship between PLAG1 expression and radiosensitivity. When analyzing the correlation between PLAG1 expression levels and gene mutations, we found that the major mutation in LGG was IDH1. Studies indicate that IDH1 plays a crucial role in the metabolic regulation of LGG epigenetics (56). The enzyme produced by IDH1 is pivotal in a key stage of the citric acid cycle; its mutation can trigger the abnormal accumulation of 2-hydroxyglutarate, resulting in the CpG island methylation phenotype and heightened histone methylation in glioma, which are deemed as critical early events in LGG (57). Malueka et al. retrospectively reviewed the clinical data of 106 LGG patients and noted that patients with IDH1 mutations experienced shorter intervals between symptom onset and initial surgical intervention, as well as longer overall survival (58). Ren et al. discovered that LGG patients with IDH1 mutations displayed enhanced infiltration of natural killer cells, which correlated with improved treatment response and prognosis (59). These findings are to some extent consistent with the study by Houillier et al., who observed that IDH mutations were associated with prolonged survival and better response to temozolomide in patients with LGG (60).

We found that PLAG1 expression was positively correlated with the expression of RNA methylation-related genes in a variety of tumors. RNA methylation plays an important role in regulating RNA stability, translation and splicing in tumor cells (61). Clinical trials are underway for targeted drugs that focus on RNA methylation (62). Studies have found that PLAG1 is regulated by RNA. In colorectal cancer, PLAG1 is a gene directly targeted by miR-181a/135a/302c, and then affects the IGF2 signaling pathway, regulates the proliferation and drug resistance of colorectal cancer cells, and affects their sensitivity to 5-FU (63). In addition, the competing endogenous RNAcircPOFUT1 can relieve the inhibitory effect of PLAG1 by binding to miR-488-3p, and PLAG1 regulates ATG12 expression to enhance tumor cell autophagy, thereby increasing the resistance of gastric cancer cells to cisplatin (44). PLAG1 silencing can inhibit the sensitivity of ovarian cancer cells to cisplatin through IGF2 signaling pathway (64). In chronic lymphocytic leukemia (CLL), multiple miRNA regulate PLAG1 expression through 3’UTR binding sites. However, increased methylation of the miRNA promoter region in CLL cells results in decreased miRNA transcription, which attenuates the repression of PLAG1 and leads to its overexpression. This may be an important mechanism in the development of CLL (65). The composition and status of the TME not only impact tumor occurrence and metastasis but also influence how tumors respond to treatment (66). We evaluated the immune scores of different cell types and found a significant positive correlation between PLAG1 expression and the abundance of immune cell infiltration in various tumors, including LGG. This suggests that PLAG1 may have the potential to predict response to immunotherapy. In addition, high expression of PLAG1 was positively correlated with multiple immune-related genes, indicating increased sensitivity to immunotherapy. In this study, the correlation between PLAG1 expression and susceptibility to multiple drugs was analyzed. These drugs include many small molecule drugs with potential clinical application. Tubastatin A, a selective histone deacetylase 6 inhibitor, has the potential to treat a variety of tumors (67). Yuan et al. found that palladium nanoparticles combined with Tubastatin A could enhance the apoptosis of breast cancer cells (68). Similarly, Li et al. demonstrated that the combination of temozolomide and Tubastatin A could induce apoptosis in drug-resistant glial tumor cells and help overcome drug resistance (69). AT13387, an inhibitor of Hsp90, is currently being explored in combination with radiotherapy for the treatment of NHSC. Studies have suggested that AT13387 causes tumor cell cycle arrest, especially in the G2/M phase, and inhibits DNA damage repair processes that are normally activated in response to irradiation (70). AT13387 has been observed to effectively reduce the expression of surface proteins, such as epidermal growth factor receptor, which are commonly associated with tumor aggressiveness and poor prognostic outcomes (71). Our findings suggest that PLAG1 could serve as a potential target gene for clinical applications.

In BLCA, GO enrichment analysis showed that the PLAG1 high expression group was significantly enriched in epidermal related processes, suggesting its potential involvement in epithelial differentiation and plasticity. In contrast, the PLAG1 low expression group was enriched in chemokine-mediated signaling pathways and immune-related KEGG pathways, suggesting that PLAG1 downregulation may be associated with enhanced BLCA immune response. Our results also suggested that PLAG1 expression was negatively correlated with immune infiltration in BLCA. Chemokine-mediated pathways are critical for recruiting immune cells into the TME, and their enrichment in the low PLAG1 group raises the possibility that PLAG1 may play an immunosuppressive role (72). PLAG1 positive co-expressed genes were mainly enriched in macroautophagy and respiratory electron transport chain, suggesting that PLAG1 may promote tumor cell survival by regulating autophagy and mitochondrial metabolism, which may also affect BLCA drug resistance.

This study has the following limitations; first, our data relied on public databases, which, while providing large-scale datasets, introduce potential data heterogeneity and batch effects. Secondly, our conclusions still need to be verified by *in vitro* and *in vivo* experiments to confirm the role of PLAG1 in cancer progression and immune regulation. Finally, despite PLAG1’s potential as a prognostic biomarker and therapeutic target, its clinical relevance remains to be confirmed in cohort studies, and future studies should focus on mechanistic studies, multi-omics data integration, and clinical validation to validate PLAG1’s role. Despite these limitations, our study sets the stage for future studies, highlighting the need for further functional and clinical studies to fully elucidate the significance of PLAG1 in tumors.

## Conclusion

PLAG1 has the potential to be a prognostic marker and a therapeutic target for cancer patients.

## Data Availability

The raw data supporting the conclusions of this article will be made available by the authors, without undue reservation.

## Supplementary material

Supplementary Figure 1 Correlation between PLAG1 expression and clinical features and analysis of differentially expressed genes in BLCA. **(A)** Correlation between PLAG1 expression and T stage; **(B)** correlation between PLAG1 expression and N stage; **(C)** correlation between PLAG1 expression and M stage; **(D)** correlation between PLAG1 expression and tumor stage; **(E)** correlation between PLAG1 expression and age; **(F)** Differentially expressed gene analysis of PLAG1 expression in BLCA.

## Glossary

ACC: Adrenocortical carcinoma
BLCA: Bladder Urothelial Carcinoma
BRCA: Breast invasive carcinoma
CESC: Cervical squamous cell carcinoma and endocervical adenocarcinoma
CHOL: Cholangiocarcinoma
COAD: Colon adenocarcinoma
READ: Rectum adenocarcinoma Esophageal carcinoma
DLBC: Lymphoid Neoplasm Diffuse Large B-cell Lymphoma
ESCA: Esophagus carcinoma
GBM: Glioblastoma multiforme
GBMLGG: Glioma
HNSC: Head and Neck squamous cell carcinoma
KICH: Kidney Chromophobe
KIPAN: Pan-kidney cohort (KICH+KIRC+KIRP)
KIRC: Kidney renal clear cell carcinoma
KIRP: Kidney renal papillary cell carcinoma
LAML: Acute Myeloid Leukemia
LGG: Brain Lower Grade Glioma
LIHC: Liver hepatocellular carcinoma
LUAD: Lung adenocarcinoma
LUSC: Lung squamous cell carcinoma
MESO: Mesothelioma
OV: Ovarian serous cystadenocarcinoma
PAAD: Pancreatic adenocarcinoma
PCPG: Pheochromocytoma and Paraganglioma
PRAD: Prostate adenocarcinoma
READ: Rectum adenocarcinoma
SARC: Sarcoma
STAD: Stomach adenocarcinoma
SKCM: Skin Cutaneous Melanoma
STES: Stomach and Esophageal carcinoma
TGCT: Testicular Germ Cell Tumors
THCA: Thyroid carcinoma
THYM: Thymoma
UCEC: Uterine Corpus Endometrial Carcinoma
UCS: Uterine Carcinosarcoma
UVM: Uveal Melanoma
PLAG1: Pleomorphic adenoma gene 1
TCGA: The Cancer Genome Atlas
HPA: Human Protein Atlas
DSS: Disease-specific survival
PFI: Progression-free interval
DNAss: DNA methylation based stemness score
DMPss: Differentially methylated probes-based stemness score
EHNss: Enhancer elements/DNA methylation-based stemness score
RNAss: RNA expression-based stemness score
EREG.EXPss: Epigenetically regulated gene expression-based stemness score
EREG-METHss: Epigenetically regulated DNA methylation-based stemness score
TMB: Tumor mutation burden
MATH: Mutant-allele tumor heterogeneity
LOH: Loss of heterozygosity
HRD: Homologous recombination deficiency
MSI: Microsatellite instability
TME: Tumor microenvironment
GO: Gene Ontology
GSEA: Gene Set Enrichment Analysis
KEGG: Kyoto Encyclopedia of Genes and Genomes
GDSC: Genomics of Drug Sensitivity in Cancer
CTRP: Cancer Therapeutics Response Portal
RT-qPCR: Real-time quantitative polymerase chain reaction
SiRNA: Small interfering RNA
HSC: Hematopoietic stem cell
CLL: Chronic lymphocytic leukemia
