# Supplementary figures and images for: Comprehensive pan-cancer analysis identifies PLAG1 as a key regulator of tumor immune microenvironment and prognostic biomarker
Source: Front Immunol. 2025 Apr 10;16:1572108. doi: 10.3389/fimmu.2025.1572108 (PMC12018345; doi:10.3389/fimmu.2025.1572108)

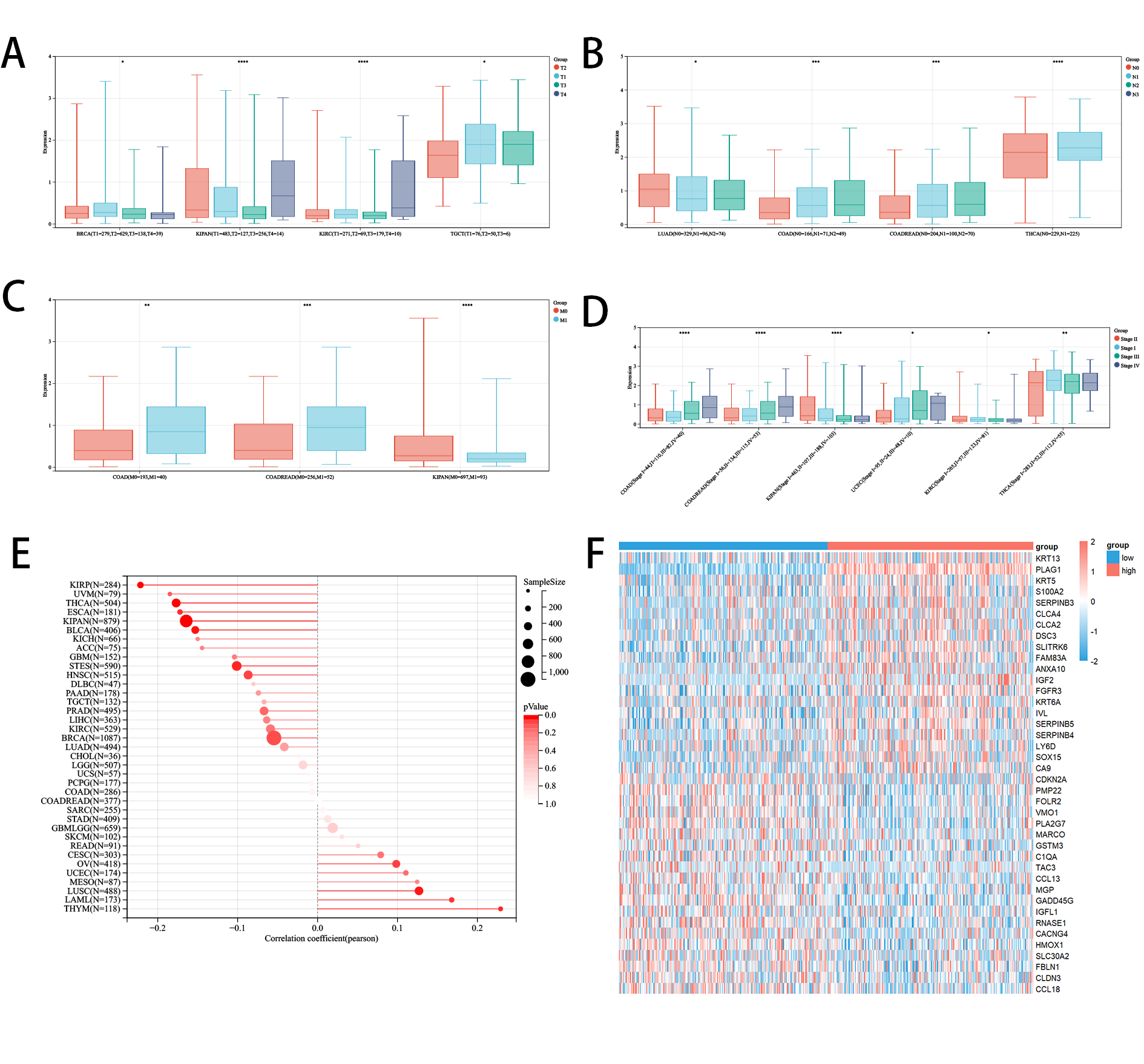

Supplement: Supplementary Figure 1 — Correlation between PLAG1 expression and clinical features and analysis of differentially expressed genes in BLCA. (A) Correlation between PLAG1 expression and T stage; (B) correlation between PLAG1 expression and N stage; (C) correlation between PLAG1 expression and M stage; (D) correlation between PLAG1 expression and tumor stage; (E) correlation between PLAG1 expression and age; (F) Differentially expressed gene analysis of PLAG1 expression in BLCA. [file Image1.tif]
